# Marek’s disease virus oncoprotein Meq physically interacts with the chicken infectious anemia virus-encoded apoptotic protein apoptin

**DOI:** 10.18632/oncotarget.25628

**Published:** 2018-06-22

**Authors:** Andrew C. Brown, Vishwanatha R.A.P. Reddy, Joshua Lee, Venugopal Nair

**Affiliations:** ^1^ Viral Oncogenesis Group, The Pirbright Institute, Pirbright, Surrey, GU24 0NF, UK; ^2^ Wellcome Trust Centre for Human Genetics, Oxford, OX3 7BN, UK; ^3^ Bristol University, Bristol, BS8 1TH, UK

**Keywords:** Meq, apoptin, MDV, CAV, lymphoma

## Abstract

Marek's disease (MD) is a neoplastic disease of poultry caused by Marek's disease virus (MDV), a highly contagious alphaherpesvirus. Meq, the major MDV oncoprotein, induces neoplastic transformation of T-cells through several mechanisms, including inhibition of apoptosis. In contrast, the chicken anemia virus (CAV)-encoded protein apoptin (VP3) is a powerful inducer of apoptosis of tumor cells, a property that is exploited for anticancer therapeutics. Although the molecular mechanisms of selective induction of tumor cell apoptosis by apoptin are not fully understood, its tumor cell–restricted nuclear translocation is thought to be important. Co-infection with MDV and CAV is common in many countries, CAV antigens are readily detectable in MD lymphomas, and the MDV-transformed T-lymphoblastoid cell lines such as MSB-1 is widely used for propagating CAV for vaccine production. As MDV-transformed cell lines express high levels of Meq, we examined here whether CAV-encoded apoptin interacts with Meq in these cells. Using immunofluorescence microscopy, we found that apoptin and Meq co-localize to the nucleus, and biochemical analysis indicated that the two proteins do physically interact. Using a combination of Meq mutagenesis and co-immunoprecipitation, we demonstrate that apoptin interacts with Meq within a region between amino acids 130 and 140. Results from the IncuCyte assay suggested that Meq inhibits apoptin-induced apoptosis activity. In summary, our findings indicate that Meq interacts with and inhibits apoptin. Insights into this novel interaction between Meq and apoptin will relevance for pathogenesis of coinfections of the two viruses and in CAV vaccine production using MDV-transformed cell lines.

## INTRODUCTION

As a rapid-onset neoplastic disease of chickens, Marek's disease (MD), caused by the highly contagious Marek's disease virus (MDV), is generally considered an excellent model for studying virus-induced T-cell lymphomas. A number of MDV-transformed T-lymphoblastoid cell lines derived from primary lymphomas have been established by different laboratories. MSB-1 is one of the first such cell lines established [1, 2], and has since been studied extensively to understand the molecular basis of MDV latency [3]. MDV establishes a latent infection in most of the cells in these cell lines, and the viral gene expression in MSB-1 is restricted to a very limited set of genes, which include the major oncoprotein Meq encoded from the transcriptionally active repeat regions of the MDV genome [4]. Meq, a basic leucine zipper (b-ZIP) transcription factor critical for MDV oncogenicity, is primarily expressed in the nucleus [5] although it can be detected in the cytoplasm during certain stages of the cell cycle [6]. Transcriptional function of Meq is dependent on its dimerization with bZIP proteins such as c-Jun, c-Fos and ATF-3 [7, 8]. Meq also has non-bZIP interactions with transcriptional co-repressor CtBP [9] and tumour suppressor protein p53 [10], and can inhibit apoptosis through the regulation of Bcl2 and p53 [10–12].

Chicken anemia virus (CAV) is a major avian pathogen associated with severe economic losses throughout the world [13]. First identified during an outbreak of MD, CAV infection leads to the development of anemia, immunosuppression and increased mortality [14]. VP1, VP2 and VP3 are three proteins encoded by CAV. The 121 amino acid-long VP3 (also known as apoptin) has been extensively studied due to its unique property of inducing cell cycle arrest and apoptosis exclusively in transformed cells [15, 16]. The ability of apoptin to induce apoptosis selectively in transformed cells, but not in normal cells, is thought to be related to its accumulation in the nucleus, since apoptin has a cytoplasmic location in untransformed cells. This is dependent on nuclear localization (NLS) and nuclear export (NES) signals present respectively at the N and C terminal of apoptin [17, 18]. The nuclear accumulation of apoptin is also be influenced by oncoproteins such as the SV40 large T-antigen [19] and Bcr-Abl [20]. It has also been shown that DNA damage response induces nuclear re-localization of apoptin in primary cells [21]. Apoptin has been shown to interact with multiple partners including HIPPI, HSP70, APC1 and Bcr-Abl, the p85 subunit of PI3-Kinase and AKT and DEDAF [20, 22–27]. The best-characterised binding motif for apoptin is the SH3 domain that is present in Bcr-Abl and p85 subunit of PI3-Kinase [20], outside of this domain an apoptin interacting motif has not been described. The induction of apoptosis by apoptin occurs in a p53-independent manner [28] and is not always affected by the anti-apoptotic Bcl-2 protein [29, 30]. It is long known that MDV-transformed T-cell lines such as MSB-1 can support the replication of CAV [31]. Similarly, experimental studies showed infection of MD lymphomas by CAV [32]. Since both MSB-1 and MD lymphomas express high levels of Meq, we examined whether there is any physical and functional interaction between apoptin and Meq.

## RESULTS

### Localization of Meq and apoptin

**Figure 1:**
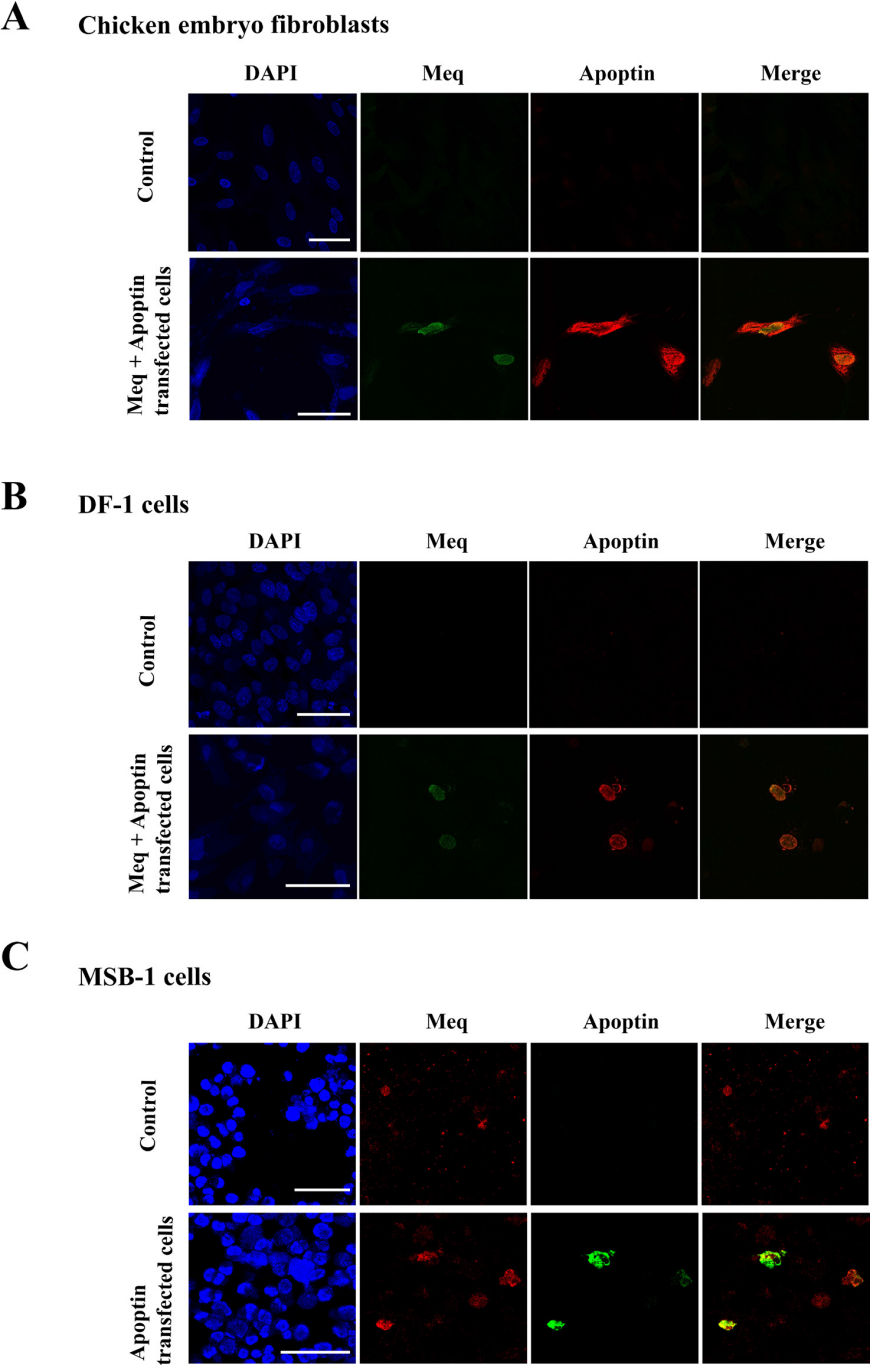
Localisation of Meq and Apoptin Immunofluorescence was carried out on (**A**) CEF and (**B**) DF-1 cells transfected with expression constructs containing wild type Meq and N-terminally FLAG tagged Apoptin. Cells were fixed after 24–48 hours and stained with polyclonal Meq (green fluorescence) or monoclonal FLAG (red fluorescence) antibodies. In both cases CEF and DF-1 cells showed localisation of both Apoptin and Meq in the nucleus of the transfected cells. (**C**) MSB-1 cells were transfected with FLAG-Apoptin, fixed and then stained for endogenous levels of Meq (red fluorescence) or FLAG tagged Apoptin (green fluorescence). In MSB-1 cells Apoptin was also localised in the nucleus along with Meq. Upper panel figures of (A), (B) and (C) were from CEF, DF-1 and MSB-1 isotype controls/cells, respectively. Confocal photomicrographs are representative of three independent experiments on CEF, DF-1 and MSB-1 cells. Scale bar represents 40 μm.

The potential physical interaction between the two proteins was investigated initially by immunofluorescence analysis in chicken embryo fibroblasts (CEF) transfected with Meq and an N-terminal FLAG-tagged apoptin (FLAG-Apoptin) expression constructs. Using specific polyclonal rabbit anti-Meq and monoclonal anti-FLAG antibodies respectively, we demonstrated nuclear co-localisation of Meq and apoptin in the transfected cells (Figure 1). While Meq expression was restricted almost exclusively to the nucleus, apoptin was distributed both in the nucleus and the cytoplasm of CEF (Figure 1A). Nuclear co-localisation of the two viral proteins was also demonstrated in transfected DF-1 cells, an immortalized chicken embryo fibroblast-derived cell line (Figure 1B). We also used immunofluorescence assay to examine whether apoptin co-localised with the endogenously expressed Meq in MSB-1 cells. MSB1 cells are known for endogenous expression of Meq protein [4]. Transfection of FLAG-tagged apoptin construct into MSB-1 showed co-localisation of endogenous Meq with transfected FLAG-tagged apoptin in the nucleus (Figure 1C).

### Interaction of Meq and apoptin

**Figure 2:**
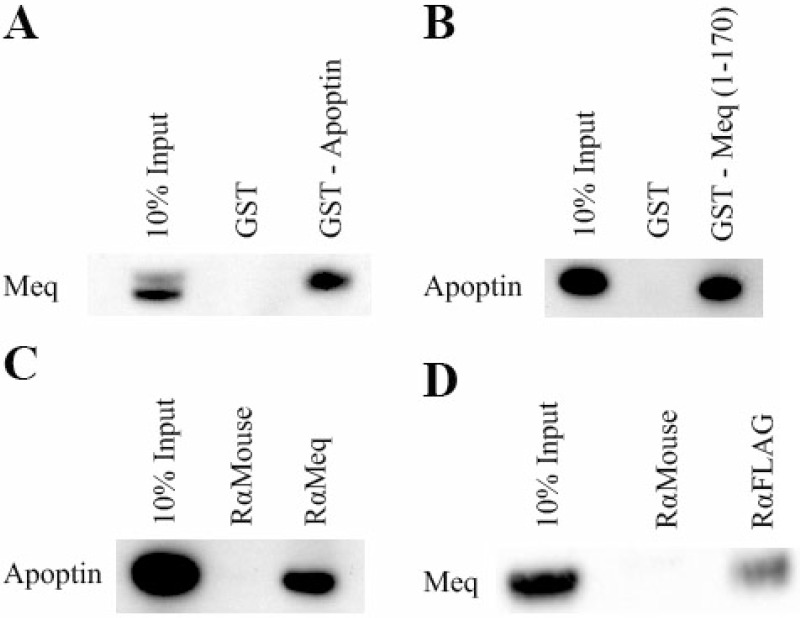
Meq and Apoptin interact Initially GST-pull-down assays were used to investigate if Meq and Apoptin interact. The pulldown assays were performed both ways. (**A**) GST-Apoptin was used against MSB-1 lysate and (**B**) GST-Meq (1–170) against induced U2OS-Apoptin lysate. Lysates were incubated with GST and GST-Meq (1–170) or GST-Apoptin. Bound protein were resolved on an SDS 4–12% Bis-Tris gel along with 10% of input protein and western blotted with Meq and monoclonal FLAG antibodies respectively. The GST pull-down showed that Meq interacts with GST-Apoptin and Apoptin interacts with GST-Meq (1–170). Immunoprecipitation was then carried out using DF-1 cells co-transfected with Meq and N-terminal tagged FLAG-Apoptin. Protein complexes were pulled out using (**C**) rabbit anti-Meq and (**D**) anti FLAG. Bound protein were resolved on an SDS 4–12% Bis-Tris gel along with 10% of input protein and western blotted with FLAG and monoclonal Meq antibodies respectively. Both Meq and Apoptin were able to pull out the other protein from the lysate demonstrating that strong interaction occur between them. GST was negative control in (A) and (B) in pull down assays. Rabbit anti-mouse was isotype control in (C) and (D) in immunoprecipitation assays. Western blot figures are representative of three independent experiments of GST-pull-down and immunoprecipitation assays of Meq and Apoptin.

GST-pull down assays were performed to obtain biochemical evidence for physical interaction between Meq and apoptin. N-terminal GST-apoptin fusion protein expressed in E. coli purified using glutathione sepharose beads and MSB-1 cell lysates that express the full length Meq protein were used in the assay. Detection of Meq in the western blots using the anti-Meq monoclonal antibody FD7 (Figure 2A) showed that GST-apoptin can pull down Meq from the MSB-1 lysate, demonstrating the interaction between the two proteins. For further confirmation of the interaction, we also carried out the reverse experiment asking whether N-terminal GST-Meq (1–170 aa) fusion protein can pull down apoptin from the cell lysates of U2OS cell line that expressed N-terminal FLAG-tagged apoptin from a doxycycline-inducible construct. Western blot analysis with monoclonal antibodies against Meq and FLAG also confirmed the interactions between apoptin and the N-terminal (1–170 aa) domain of Meq (Figure 2B).

For the further confirmation of the interaction of the two proteins, immunoprecipitation assays were performed using the same expression constructs used in the immunofluorescence assay. Briefly, DF-1 cells were transfected with the Meq and FLAG-apoptin expression constructs. After 24–48 hours, transfected cells were lysed, pre-cleared and complexes precipitated using the appropriate antibody and protein-G sepharose beads before western blot analysis using specific antibodies. Specific reciprocal immunoprecipitation of apoptin-Meq complexes using Meq (Figure 2C) and FLAG (Figure 2D) antibodies provided further evidence for biochemical interactions of the two proteins.

### Apoptin interaction occurs between 130 and 140 amino acid residues of Meq

**Figure 3:**
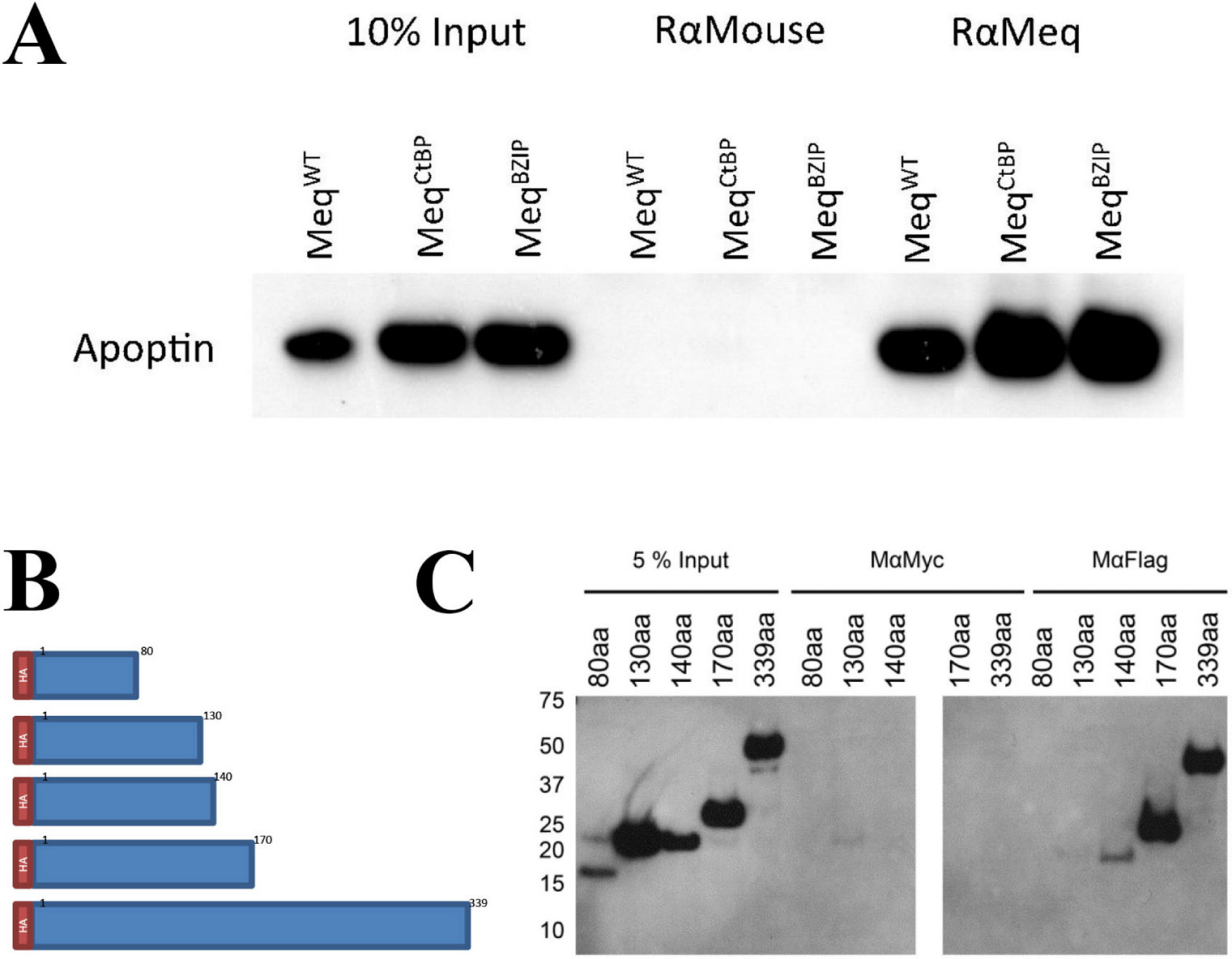
Meq and Apoptin do not interact through Meq-CtBP or Meq-BZIP motifs, but interact between 130 and 140 amino acids of Meq (**A**) Immunoprecipitation was carried out using DF-1 cells co-transfected with Meq, Meq-CtBP or Meq-BZIP mutants and N-terminally tagged FLAG-Apoptin. Protein complexes were pulled out using (Figure 2C) rabbit anti-Meq and (Figure 2D) anti FLAG. Bound protein were resolved on an SDS Bis-Tris gel along with 10% of input protein and western blotted with FLAG antibodies. Meq was able to pull out Apoptin from the lysate in all cases showing the CtBP motif and leucine zipper were not crucial for the interaction. (**B**) Four C-terminally truncated Meq proteins of the first 80, 130, 140 and 170 amino acids with an N-terminal HA tag were constructed to localise the Apoptin interaction domain within Meq. (**C**) The four truncations of Meq along with the wild type were used in immunoprecipitation assays using DF-1 cells co-transfected with N-terminally tagged FLAG-Apoptin. Protein complexes were pulled out using mouse anti-Meq and anti-FLAG. Bound protein were resolved on an SDS Bis-Tris gel along with 10% of input protein and western blotted with HA-HRP direct conjugate antibodies. Meq was able to pull out Apoptin from the lysate as long as the N-terminal 140 aa were present, the N-terminal 80 or 130 amino acids were not sufficient. Suggest the interaction occurs between amino acids 130 and 140. Rabbit anti-mouse was isotype control in (A) and mouse anti-Myc was isotype control in (C). Western blot images are representative of triplicate experiments.

Immunoprecipitation assays were also used to identify the apoptin-interacting domain within Meq protein. For this, we initially used Meq constructs used to demonstrate the interactions of Meq with CtBP and BZIP-domain containing proteins [9, 33]. Expression constructs of Meq-CtBP with a mutation in the CtBP-binding motif or Meq-BZIP with a non-functional leucine zipper motif [9, 33] were co-transfected into DF-1 cells along with FLAG-apoptin construct and complexes precipitated using polyclonal rabbit anti-Meq antibodies. Detection of precipitated complexes in Western blotting assay with monoclonal FLAG antibody demonstrated that both these mutations did not affect the interaction of apoptin with Meq (Figure 3A). In order to identify the putative apoptin-interacting domains within Meq, we then generated N-terminal HA tagged full length Meq (1–339) and a series of C-terminal truncated Meq constructs (1–80, 1–130, 1–140 and 1–170 amino acid residues) in pcDNA3.1 vector (Figure 3B). Each of these Meq-expression constructs were co-transfected with FLAG-apoptin plasmid into DF-1 cells and protein complexes immunoprecipitated with anti-Meq or anti-FLAG antibodies. SDS-PAGE-resolved immunoprecipitates and were subjected to Western blot analysis with HA-tagged antibody. The ability of the N-terminal 140, 170 and the full length Meq constructs, but not the N-terminal 80 or the 130 constructs, to pull down apoptin indicated that the potential interaction of apoptin occurs between amino acids 130 and 140 of Meq (Figure 3C).

### Meq inhibits apoptin in DF-1 cells

**Figure 4:**
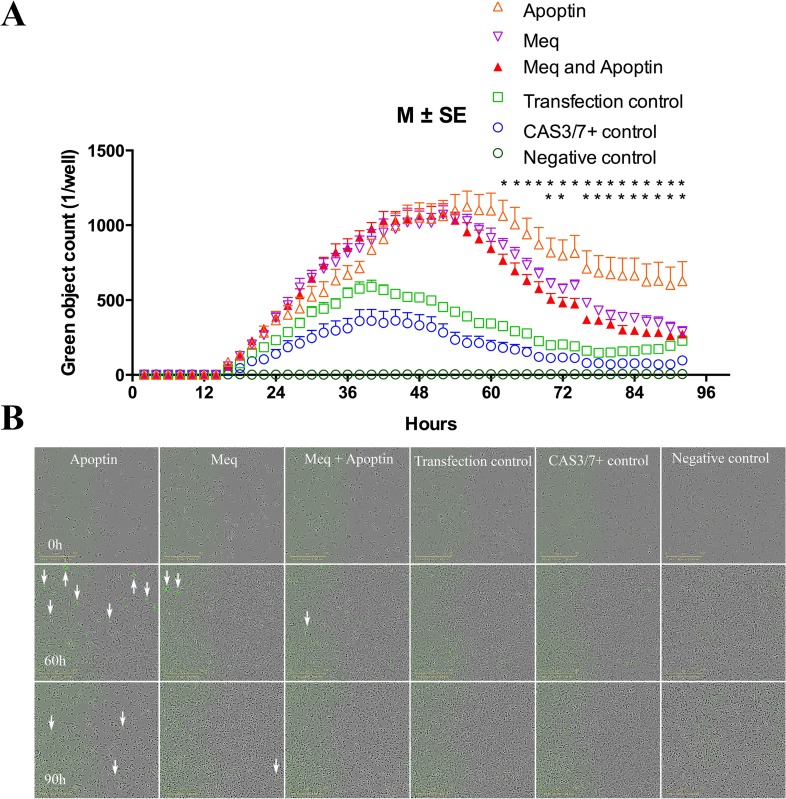
Meq, Apoptin, Meq-Apoptin plasmid transfected DF-1 cells were monitored in real time by the live cells imaging system IncuCyte (**A**) Meq-Apoptin co-transfected cells apoptosis was significantly lower than Apoptin transfected cells between 60 and 92 h. And Meq-transfected cells apoptosis was significantly lower compared to Apoptin-transfected cells between 68 and 92 h, except at 74 h. Growth curves are shown as means of six independent experiments ± standard error (SE). An asterisk (^*^) indicates statistically significant difference (*P* < 0.05) between Apoptin and Meq-Apoptin or Meq transfected cells. (**B**) Representative IncuCyte live cells images illustrating evolution of apoptotic cells (green) at 0, 60 and 90 h. Transfection control, Caspase 3/7 positive control and negative control cells were used as controls.

We next wanted to investigate the functional significance of Meq-apoptin interactions. With apoptin functioning as a strong inducer of apoptosis, and Meq known for its apoptosis-inhibitory function [10–12], we examined the effects of their interactions on apoptosis in the presence of Caspase 3/7 Apoptosis Assay Reagent. IncuCyte ZOOM Live-Cell Imaging system was used for kinetic monitoring of apoptosis on DF-1 cells transfected with the two proteins individually or together. Fluorescent images collected at every 2 hour intervals. Quantitation of apoptosis from the fluorescent images collected at every 2 hour intervals showed that apoptosis was significantly lower between 60 and 92 h in the Meq-apoptin co-transfected cells than in the cells transfected only with apoptin (Figure 4A). As shown in Figure 4A, the levels of apoptosis was also significantly lower in the Meq-transfected cells compared to the apoptin-transfected cells between 68 and 92 h, except at 74 h (M ± SE, *n* = 6).

The kinetics of apoptotic cells at 0, 60, and 90 h are illustrated in Figure 4B. Supplementary videos 1 to 6 represent the apoptotic cells kinetics between 0 and 92 h of Apoptin, Meq, Meq-Apoptin, Transfection control, Caspase 3/7 control and negative control cells, respectively. The phase confluence of cells was inversely related to apoptosis (green object count) of cells, and Meq-transfected cells (either alone or when co-transfected with apoptin) showed significantly higher phase confluence than apoptin transfected cells after 50 h (data not shown). This data demonstrating the inhibitory effect of Meq on the apoptosis-inducing function was also confirmed independently using xCELLigence [34] system (data not shown).

Further, to understand the significance of the 130–140 amino acid region of Meq, a synthetic peptide (Cambridge peptides, Birmingham, UK) mimicking the region (YPYDVPDYA-LTVTLGLLTTP; HA-tagged to N-terminal of 130–140 amino acids of Meq) was evaluated for its ability to inhibit Meq-apoptin interactions in DF-1 cells. Meq synthetic peptide 130–140 was transfected into DF-1 cells by using the Chariot Transfection system, according to the manufacturer instructions (Active motif, Rixensart, Belgium). β-galactosidase was used as positive control for the Chariot Transfection reagent (Active motif, Rixensart, Belgium). To analyze the functional effects of the synthetic peptide 130–140, the number of Meq and apoptin co-localized cells were evaluated in the presence or absence of synthetic peptide by using a confocal microscope. Ten randomly selected regions were counted in each condition for three independent experiments. We observed that there was no change in number of apoptin and Meq co-localized cells in presence (1.70 ± 1.25%) or absence (2 ± 1.25%) of Meq synthetic peptide 130–140 (M ± SD, *n* = 3).

## DISCUSSION

MD is a good model for studying virus-induced T-cell lymphomas. Meq, the major oncoprotein of MDV, induces neoplastic transformation of T cells through several mechanisms, including inhibition of apoptosis [10–12, 35]. As a transcriptional regulator, Meq has a nuclear distribution and can function as a Meq/Meq homodimer or a heterodimer with a number of other cellular BZIP proteins. In the past, we reported that Meq interacts with CtBP via the proline-leucine-aspartic acid-leucine-serine (PLDLS) motif, an interaction critical for induction of lymphomas [9]. Similarly, Meq homodimerization was shown to be essential for the induction of T-cell lymphoma by MDV [33].

CAV is an economically important virus of chickens, causes severe anemia, immunosuppression, lymphoid atrophy and mortality [14, 32]. CAV replicates in MDV-transformed T-cells and MDV-CAV coinfection results in increased pathogenesis, despite the molecular interactions are remain poorly understood [14, 32, 36]. Although a number of interacting partners of Meq or apoptin have been identified, [9, 33, 37–40], it is still unclear whether Meq and apoptin proteins do directly interact. Here, we report for the first time that Meq protein can physically interact with apoptin protein, with potential functional significance.

Data of immunofluorescence staining, GST pull-down, and immunoprecipitation assays demonstrated that Meq physically interacts with apoptin, and interaction was shown to be in a region between amino acids 130 and 140 of Meq. The CtBP and BZIP-domains are not involved in interaction with apoptin. IncuCyte ZOOM Live-Cell Imaging and xCELLigence (data not shown) assays were used to examine the functional significance of Meq-apoptin interaction in DF-1 cells. Observations on the inhibition of apoptosis function by Meq have corroborated the functional significance of the interactions of these proteins, with potential roles in MDV and CAV pathogenesis in co-infected chickens. The inhibition of apoptin activity by Meq could be crucial in T-lymphoblastoid transformation of MDV, and may be further helpful for sustenance and dissemination of CAV [32, 36]. There have been several reports of viruses modulating the cell microenvironment for their own advantages [10, 17, 23, 41–47]. Indeed, both Meq and apoptin modulate the cell cycle machineries [17, 23, 47] and maintenance of favorable cellular conditions for viral replication is strategically important during co-infections with the two viruses [45, 46, 48]. Further studies on CD4^+^ and CD8^+^ T cells populations of lymphomas collected from co-infected chickens will help to understand the role and significance of these complex interactions in co-pathogenesis of CAV and MDV [32, 36]. Further insights into the Meq-apoptin interactions are also valuable to examine the interactions of apoptin with other oncoproteins such as EBNA3A/C of Epstein-Barr virus or EVI1 of myeloid leukaemia [49, 50], because of their interactions with CtBP [9, 50].

As mentioned earlier, apoptotic function of apoptin in transformed cells is reliant on its translocation to the nucleus [17, 18]. Although we have observed co-localisation of Meq and apoptin in the nucleus of transfected fibroblasts and in MSB-1 cells (Figure 1), we don't know whether Meq and its nuclear localisation signals are directly involved in the active nuclear translocation of apoptin. Nevertheless, based on the interaction of the two proteins, one could suggest the possibility that Meq may activate apoptin at threonine 108 [51] through a Meq-regulated cellular kinase in transformed cells as was suggested in the case of SV40 large-T antigen [19]. In support of this hypothesis, altered expression of several kinases has been demonstrated in cells overexpressing Meq [12].

Apoptin is known to bind multiple partners including Hippi [22, 23], APC1 [23], HSP70 [24], Crm1 protein for nuclear export [17], Bcr-Abl oncoprotein [20], p85 subunit of PI3-Kinase [25, 26], DEDAF (human death effector domain-associated factor) [27], FADD, Bcl 10 [52], and PKC (protein kinase C) beta [39]. Recent studies have shown that apoptin inhibits Bcr-Abl1 kinases in chronic myeloid leukemia (CML), and regulates the activity of protein kinase C (PKC) in myeloma cell lines, respectively [39, 40]. However, none of these proteins have demonstrated a conserved interaction motif, although the larger SH3 domain of Bcr-Abl1 and PI3-Kinase appear to be involved [20, 25, 26]. Our studies using truncated Meq constructs have demonstrated the importance of sequences between 130 and 140 (LTVTLGLLTTP) of Meq in interacting with apoptin (Figure 3B and 3C). Although we examined the significance of this interacting region (130–140 aa) of Meq with a synthetic peptide, we could not demonstrate changes in the co-localization of the two proteins in the presence of the peptide in DF-1 cells. It might be that the N-terminal nuclear localization signal that transports Meq into nucleus may also be important for the interaction [5, 17] and the absence of the nuclear localization signal might explain the lack of the biological activity of this peptide [53]. Alternatively, aggregation of the protein in these cells may inhibit its effectiveness [53, 54].

Homo/hetero dimerization of Meq and multimerization of apoptin are important characteristics that are associated with the biological functions of these proteins [17, 33, 55], possibly by providing additional interface (s) for binding proteins. Previous studies have shown that N and C-terminal regions of apoptin have important cell killing (apoptotic) activity [18] and it is likely that Meq might be interacting with these N and C-terminal regions of apoptin to inhibit the apoptotic activity.

Other authors have reported that the biological functions of Meq and apoptin are modulated by their interactions with multiple protein partners [9, 22–26, 33, 35, 56]. During MDV-CAV co-infection also, there should be multiple molecular partners that would potentially interact with the Meq-apoptin complexes. Next step will be to identify the Meq interaction domains of apoptin that contribute to the inhibition of apoptosis. We will also aim to resolve the Meq-apoptin interactome to know their roles in MDV-CAV co-pathogenesis.

In summary, we have shown that Meq interacts with apoptin, and inhibits apoptin activity. Further studies in lymphoblastoid cell lines or chickens are needed to get functional insights into MDV and CAV co-pathogenesis for T-cell transformation and persistence and dissemination of CAV.

## MATERIALS AND METHODS

### Cells

Primary chicken embryo fibroblasts (CEF) were collected from 10-day-old embryonated specific pathogen free (SPF) eggs [57]. CEFs were used for immunofluorescence assay. DF1 cells are continuous cell lines of EV-0 chicken embryo fibroblasts [58]. DF-1 cells were used for immunofluorescence and immunoprecipitation assays. MSB-1 lymphoblastoid cells are T-lymphocyte cell line, and are grown in suspension [1, 2]. MSB-1 cells were used for immunofluorescence and GST pull-down assays. A human osteosarcoma cell line (U2OS) was used for GST pull-down assay.

### Plasmids

Plasmid pcDNA3.1-Meq full length (339 aa), Meq^CtBP^ and Meq^BZIP^ expression constructs were used and described previously [9, 33]. Plasmid pcDNA3.1 Flag tagged full length apoptin (FLAG-Apoptin) and pRTS apoptin constructs were generated by PCR. The C-terminal truncated Meq constructs (1–80, 1–130, 1–140 and 1–170 aa residues) were generated in order to identify the putative apoptin interacting domains within Meq.

### Plasmids transfection

CEFs (1 × 10^6^) and DF-1 cells (1 × 10^6^) were plated in a six-well culture plates. Meq and N-terminal FLAG tagged apoptin expression constructs were transfected into CEFs by Lipofectamine and DF-1 cells by Lipofectamine 2000 reagents respectively (Invitrogen, Karlsruhe, Germany). CEFs were 70–80% confluence and DF-1 cells were 90–95% at the time of transfection. MSB-1 cells (1 × 10^6^) were transfected with plasmid Flag tagged apoptin by electroporation (Amaxa Biosystems, Nucleofector, Koln, Germany), and was used for immunofluorescence staining and GST-pull down assay. An inducible human osteosarcoma (U2OS)-apoptin cell line was generated by plasmid pRTS apoptin transfection into U2OS cells, and subsequently selected with puromycin (1 μg/mL). Apoptin protein expression in U2OS cells was induced by doxycycline (1 μg/mL), and U2OS-apoptin lysate was used for GST-pull down assay. The experiments were performed in triplicate.

### Immunofluorescence

CEF, DF-1 cells and MSB-1 cells were fixed in 4% paraformaldehyde (30 min, Room temperature (RT)) and permeabilized with 0.1% Triton X-100 (15 min, RT). Then, cells were blocked in 5% bovine serum albumin (BSA) in phosphate-buffered saline (PBS) for 30 min. Cells were first stained for Apoptin (anti-Flag, clone M2) and next for Meq proteins. Cells were incubated (1 h, 37°C) with mouse monoclonal anti-Flag antibodies (1:4000 in 5% BSA), after which cells were washed three times. Then, cells were incubated (1 h, 37°C) simultaneously with Alexa 568 (CEF and DF-1 cells)/488 (MSB-1 cells)-conjugated goat anti-mouse (1:200 in 5% BSA) and rabbit polyclonal anti-Meq antibodies (1:5000 in 5% BSA). Subsequently, after washing, cells were incubated with Alexa 488 (CEF and DF-1 cells)/ 568 (MSB-1 cells)-conjugated goat anti-rabbit antibodies. Finally, after washing, cells were stained (10 min, RT) with DAPI (1:10000) and viewed by using a Leica (Wetzlar, Germany) TCS SP2 confocal laser-scanning microscope.

### GST pull-down, immunoprecipitation and Western blotting

These assays were performed in three independent experiments, essentially as described previously [50]. The antibodies used were rabbit anti-Meq (raised against GST-Meq-wild type [9], at the Institute for Animal Health, Compton, United Kingdom), rabbit-anti-Mouse (Dako, Glostrup, Denmark), mouse-anti-Myc (Santa Cruz, California, USA), rabbit-anti-FLAG (BD Biosciences PharMingen, San Diego, California, USA) and mouse-anti-Flag (Sigma Aldrich, Saint Louis, Missouri, USA).

### IncuCyte apoptosis assay

The functional interactions between Meq and apoptin proteins were determined using caspase 3/7 apoptosis assay, and experiment monitored by IncuCyte ZOOM live cell imaging (Essen Bioscience, Michigan, USA). Briefly, Meq, apoptin and Meq-apoptin plasmids transfected DF-1 cells were seeded at 5000 cells per well in a 96 well plate (Corning). After overnight incubation, caspase 3/7 reagent (1:1000) was added on cells (Essen Bioscience). Images were captured every 2 h for 92 h from four separate regions per well using a 10× objective. Green object count per well was quantified at each time point of Meq, apoptin, Meq-apoptin, transfection control, caspase 3/7 positive control and negative control cells. IncuCyte data was analysed by one-way analysis of variance (ANOVA) with Tukey *post hoc* comparisons using GraphPad Prism version 7.01 (GraphPad Software, Inc., San Diego, CA). The results were shown as mean ± standard error (SE) for six independent experiments. *P* values of < 0.05 were considered to be significant.

## SUPPLEMENTARY MATERIALS VIDEOS















## Abbreviations

CEF: chicken embryo fibroblasts
CAV: chicken anemia virus
MDV: Marek's disease virus
b-ZIP: basic leucine zipper
CtBP: Carboxyl-terminal binding protein
NLS: nuclear localization signal
NES: nuclear export signal
DEDAF: death effector domain associated factor
PKC: protein kinase C
CML: chronic myeloid leukemia
U2OS: human osteosarcoma cell line
SPF: specific pathogen free
BSA: bovine serum albumin
RT: room temperature
PBS: phosphate-buffered saline
