# Supplementary material for: Marek’s disease virus oncoprotein Meq physically interacts with the chicken infectious anemia virus-encoded apoptotic protein apoptin
Source: Oncotarget. 2018 Jun 22;9(48):28910–20. doi: 10.18632/oncotarget.25628 (PMC6034753; doi:10.18632/oncotarget.25628)
Supplement: Supplementary file 1 [file oncotarget-09-28910-s001.pdf]

Marek's disease virus oncoprotein Meq physically interacts with the chicken infectious anemia virus-encoded apoptotic protein apoptin

SUPPLEMENTARY MATERIALS

Supplementary Video 1: Apoptin transfected cells apoptosis kinetics. Apoptin transfected cells apoptosis was significantly higher than Meq-Apoptin transfected cells between 60 and 92 h (day 2, 12 hours and day 3, 20 hours) post transfection. See Supplementary_Video_1

Supplementary Video 2: Meq transfected cells apoptosis kinetics. Meq transfected cells apoptosis was significantly lower than Apoptin transfected cells between 68 and 92 h (day 2, 20 hours and day 3, 20 hours) post transfection. See Supplementary_Video_2

Supplementary Video 3: Meq and apoptin co-transfected cells apoptosis kinetics. Meq-Apoptin co-transfected cells apoptosis was significantly lower than Apoptin transfected cells between 60 and 92 h (day 2, 12 hours and day 3, 20 hours) post transfection. See Supplementary_Video_3

Supplementary Video 4: Transfection control apoptosis kinetics. Negligible apoptosis was observed compared to Apoptin, Meq and Meq-Apoptin transfected cells. See Supplementary_Video_4

Supplementary Video 5: Caspase 3/7 positive control apoptosis kinetics. Negligible apoptosis was observed compared to Apoptin, Meq and Meq-Apoptin transfected cells. See Supplementary_Video_5

Supplementary Video 6: Negative control apoptosis kinetics. Apoptosis was not observed. See Supplementary_Video_6
